# YebC, a putative transcriptional factor involved in the regulation of the proteolytic system of *Lactobacillus*

**DOI:** 10.1038/s41598-017-09124-1

**Published:** 2017-08-17

**Authors:** Lucía Brown, Josefina M. Villegas, Mariano Elean, Silvina Fadda, Fernanda Mozzi, Lucila Saavedra, Elvira M. Hebert

**Affiliations:** 1 Centro de Referencia para Lactobacilos (CERELA-CONICET), Chacabuco 145, 4000 San Miguel de Tucumán, Argentina; 2 Instituto Superior de Investigaciones Biológicas (INSIBIO), CONICET-UNT, San Miguel de Tucumán, Argentina

## Abstract

The proteolytic system of *Lactobacillus* plays an essential role in bacterial growth, contributes to the flavor development of fermented products, and can release bioactive health-beneficial peptides during milk fermentation. In this work, a genomic analysis of all genes involved in the proteolytic system of *L*. *delbrueckii* subsp. *lactis* CRL 581 was performed. Genes encoding the cell envelope-associated proteinase, two peptide transport systems, and sixteen peptidases were identified. The influence of the peptide supply on the transcription of 23 genes involved in the proteolytic system of *L*. *delbrueckii* subsp. *lactis* was examined after cell growth in a chemically defined medium (CDM) and CDM supplemented with Casitone. *prtL*, *oppA*_1_, *optS*, *optA* genes as well as o*ppDFBC* and *optBCDF* operons were the most highly expressed genes in CDM; their expression being repressed 6- to 115-fold by the addition of peptides. The transcriptional analysis was confirmed by proteomics; the up-regulation of the PrtL, PepG, OppD and OptF proteins in the absence of peptides was observed while the DNA-binding protein YebC was up-regulated by peptides. Binding of YebC to the promoter region of *prtL*, *oppA*_1_, and *optS*, demonstrated by electrophoretic mobility shift assays, showed that YebC acts as a transcriptional repressor of key proteolytic genes.

## Introduction

*Lactobacillus* (*L*.) *delbrueckii* subsp. *lactis* is a homofermentative thermophilic lactic acid bacterium (LAB) widely used as starter culture in dairy fermentation processes, such as fermented sour milks and Swiss- and Italian-type cheeses. Comparative genomic analysis of lactobacilli revealed that amino acid biosynthetic pathways are deficient to a different extent in LAB, resembling the adaptation to specific-niches^1, 2^. For instance, dairy LAB such as *L*. *delbrueckii* subsp. *lactis*, *L*. *delbrueckii* subsp. *bulgaricus* and *L*. *helveticus* have lost the majority of their amino acid biosynthetic genes^3, 4^, and therefore, depend on the use of exogenous nitrogen sources for optimal growth^5, 6^. As the concentration of amino acids and small peptides in milk is very limited^7^, the proteolytic system of LAB is crucial to obtain essential amino acids from caseins during growth in this substrate, thereby ensuring a successful fermentation. This proteolytic system consists of (i) an extracellular cell envelope-associated proteinase (CEP) that hydrolyzes caseins into oligopeptides; (ii) specialized transport systems that take up peptides into the cell; and (iii) several intracellular peptidases, which degrade peptides into amino acids^2, 8, 9^. Furthermore, this proteolytic system contributes to the flavor and texture development of fermented products^9, 10^, and may release bioactive health-beneficial peptides during milk fermentation^8–11^.

Among LAB, the proteolytic system of *Lactococcus* (*Lc*.) has received considerably more attention than that of *Lactobacillus* ^2, 9^. However, in the last years the proteolytic system of lactobacilli has gained relevance because of their ability to generate bioactive peptides from casein during milk fermentation processes^8, 10^. *L*. *delbrueckii* subsp. *lactis* CRL 581, a strain isolated from a homemade Argentinian hard cheese, possesses a CEP (PrtL) that is able to release a series of bioactive health-beneficial peptides (i.e., anti-inflammatory, antihypertensive, and phosphopeptides) from α- and β-caseins^11, 12^. It was observed that the production of bioactive peptides by PrtL was repressed by the peptide content of the growth medium^7^. In *Lc*. *lactis*, the transcriptional regulator CodY is responsible for repression of several genes (*prtP/prtM*, *opp-pepO1*, *pepD*, *pepN*, *pepC*, and *pepX*) of the proteolytic system in response to branched-chain amino acid (BCAA) availability^13–15^. However, no CodY-like homologous in lactobacilli has been found and the information about the regulation of several genes encoding proteins from their proteolytic system remains unclear.

Considering the industrial importance of the proteolytic system of *Lactobacillus* and the health-promoting attributes of the CRL 581 strain in the development of novel functional foods, a deeper insight on the functionality and regulation of the proteolytic system of *L*. *delbrueckii* subsp. *lactis* is essential to fully understand the production and processing of bioactive peptides. Therefore, we aimed to perform a genomic analysis of all genes involved in the proteolytic system of *L*. *delbrueckii* subsp. *lactis* CRL 581 and to study their transcriptional response to a peptide supply. In addition, the putative role of the YebC DNA-binding protein in the regulation of the proteolytic system of *L*. *delbrueckii* subsp. *lactis* was studied. This report correlates transcriptional analysis and proteomics information of *L*. *delbrueckii* subsp. *lactis* to better understand the regulation of the proteolytic system in this genus. Such an integrated approach can be used to predict the proteolytic potential of *Lactobacillus* to expand the knowledge on specific features likely related to its industrial applications.

## Results

### Genomic analysis of the proteolytic system

**Figure 1:**
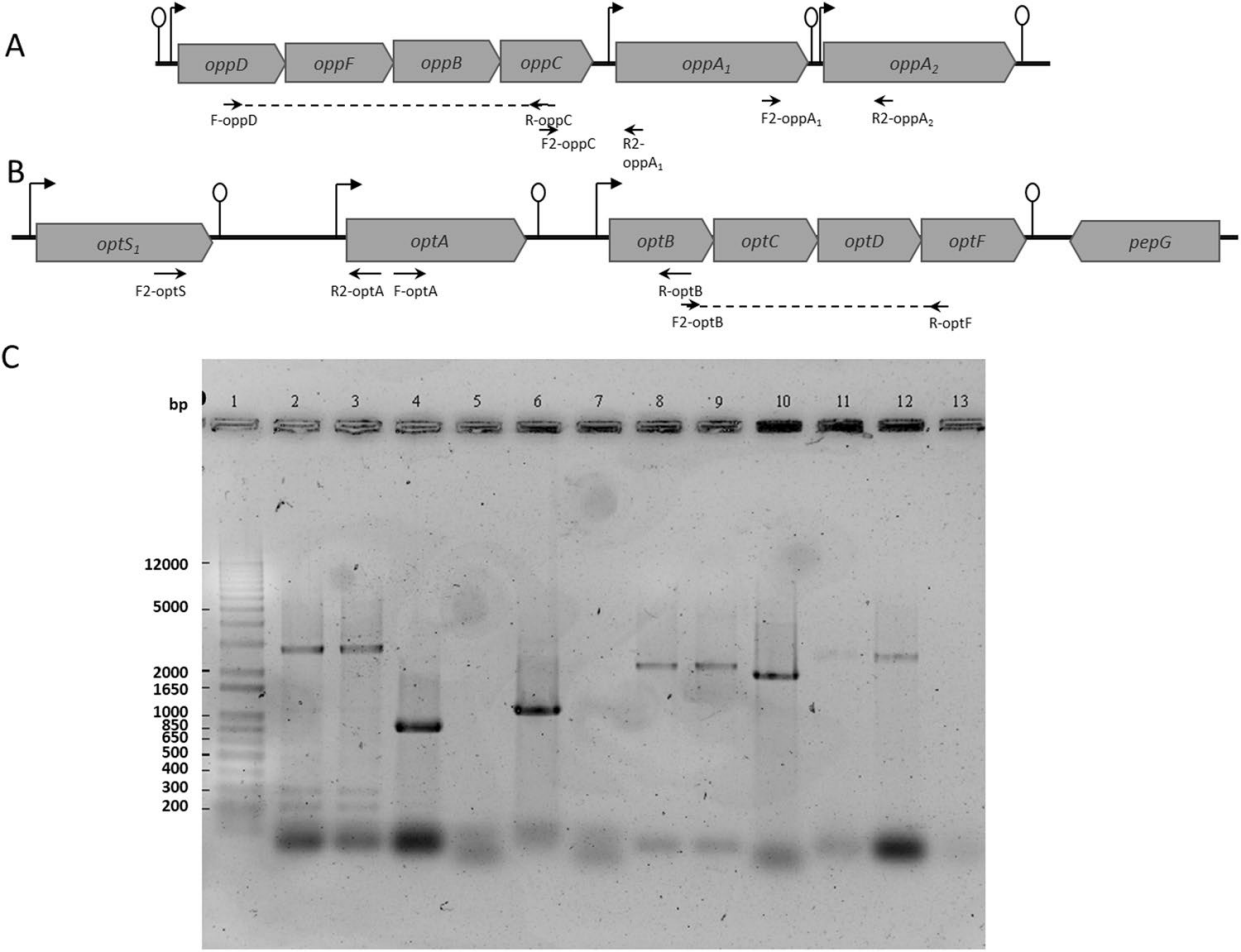
Genetic organization of genes encoding the oligopeptide transport systems (Opp and Opt) of *L*. *delbrueckii* subsp. *lactis* CRL 581. Schematic representation of the genetic organization of (**A**) *opp* genes and (**B**) *opt* genes. (**C**) RT-PCR analysis of the transcriptional organization of *opp* and *opt* operon. Broken arrows and lollipops represent the putative promoters and terminators, respectively. Dotted lines indicate the expected size of the PCR fragments. Couples of horizontal arrows represent couples of primers used for the PCR reactions, F-oppD/R-oppC (lanes 2 and 3), F2-oppC/R2-oppA_1_ (lanes 4 and 5), F2-oppA_1_/R2-oppA_2_ (lanes 6 and 7), F2-optB/R-optF (lanes 8 and 9), F2-optS/R2-optA (lanes 10 and 11) and R-optA/R-optB (lanes 12 and 13). Lane 1, molecular weight marker (1 kb Plus DNA Ladder, Thermo Fisher Scientific); lanes 2, 4, 6, 8, 10 and 12, positive genomic DNA controls; and lanes 3, 5, 7, 9, 11 and 13, PCR amplification products obtained from the cDNA generated by the RT reaction.

The genome of *L*. *delbrueckii* subsp. *lactis* CRL 581 was sequenced and only one gene encoding a CEP, named PrtL was detected^11, 16^. In contrast to *Lc*. *lactis*, no *prtM* homologous in regions flanking *prtL* has been identified. The genome data analysis revealed the presence of two oligopeptide ABC transporters, the *oppDFBCA* and the *optABCDF* operons (Fig. 1). The *oppDFBCA* operon encodes two ATP-binding proteins (OppD and OppF), two membrane proteins (OppB and OppC) and a substrate-binding protein (OppA_1_, Fig. 1A). Interestingly, an *oppA*_2_ gene, encoding an additional peptide-binding protein was found downstream of *oppA*_1_. The Opt system, represented by the *optABCDF* operon, is composed of five proteins which display identity with an oligopeptide-binding lipoprotein (OptA), two transmembrane proteins (OptB and OptC), and two cytoplasmic ATPases (OptD and OptF) (Fig. 1B). The gene order of *opt* operon is quite different from the *opp* operon (*oppDFBCA*). The *optS* gene encodes a second binding protein OptS, which is located upstream of the *optABCDF* operon and it is transcribed in the same direction as the *opt* operon (Fig. 1B). The *dtpT* gene, encoding the proton motive force (PMF)-driven dipeptide/tripeptide DtpT^17^, present in *Lc*. *lactis*, was not found in the genome of *L*. *delbrueckii* subsp. *lactis* CRL 581^2^.

Additionally, sixteen putative peptidases were detected when the genome of *L*. *delbrueckii* subsp. *lactis* CRL 581 was analyzed by RAST^18^ and submitted to the online blasting tool on MEROPS peptidase database (Table 1)^19^. Several peptidases belong to the same large protein superfamilies. For instance, aminopeptidase PepC together with endopeptidases PepG/E belong to MEROPS peptidase family C1; proline peptidases PepI, PepR and PepL belong to MEROPS family S33; aminopeptidase PepM together with proline peptidases PepP and PepQ belong to MEROPS family M24; and tripeptidases PepV and PepT belong to MEROPS family M20 (Table 1). MEROPS peptidase family C, M and S are cysteine, serine, and metallo peptidases, respectively.

**Table 1:** Peptidases of the proteolytic system of *L*. *delbrueckii* subsp. *lactis* CRL 581.

Peptidase	Gene (locus)	MEROPS Family	Substrate	Predicted molecular mass (kDa)	Isoelectric point
**Aminopeptidases**					
Aminopeptidase C	*pepC* (G134_RS08320)	C1	X↓(X)n	50.96	4.71
Lysine aminopeptidase	*pepN* (G134_RS03335)	M1	X↓(X)n	95.30	4.48
Glutamine aminopeptidase	*pepA* (G134_RS00305)	M42	E/D↓(X)n	40.01	4.51
Methionine aminopeptidase	*pepM* (G134_RS00100)	M24A	M↓(X)n	30.75	4.49
**Endopeptidases**					
Oligopeptidase F	*pepF* (G134_RS03600)	M3	(X)n↓(X)n	68.34	4.44
Oligopeptidase O	*pepO* (G134_RS01630)	M13	(X)n↓(X)n	72.02	4.45
Peptidase G	*pepG/E* (G134_RS01440)	C1	(X)n↓(X)n	49.97	4.89
**Dipeptidase**					
Dipeptidase A	*pepD (G134_RS03315)*	C69	X↓X	36.96	10.64
**Tripeptidases**					
Tripeptidase V	*pepV* (G134_RS08405)	M20A	X↓X-X	51.96	4.31
Tripeptidase T	*pepT* (G134_RS01035)	M20B	X↓X-X	47.73	4.55
**Prolyl peptidases**					
Aminopeptidase P	*pepP* (G134_RS05655)	M24B	X↓P-(X)n	40.29	4.71
Prolidase	*pepQ* (G134_RS07685)	M24B	X↓P	41.08	4.63
X-Prolyl-dipeptidyl aminopeptidase	*pepX* (G134_RS05545)	S15	X-P↓(X)n	88.42	4.91
Proline dipeptidase	*pepR* (G134_RS08875)	S33	P↓X	34.78	4.81
Proline aminopeptidase	*pepL* (G134_RS06955)	S33	P↓X	34.72	4.77
Proline aminopeptidase	*pepI* (G134_RS08815)	S33	P↓X-(X)n	32.91	5.01

### Transcriptional organization of the components of the proteolytic system

*In silico* analyses of the proteolytic system of *L*. *delbrueckii* subsp. *lactis* CRL 581, using bacterial promoter algorithms (http://www.softberry.com) suggested that *prtL* and all peptidases encoding genes are monocistronically transcribed. Furthermore, *in silico* analyses of the *opp* cluster identified putative promoters in the 5′ region of *oppD*, *oppA*_1_, and *oppA*_2_ genes, and putative terminators downstream of *oppA*_1_ and *oppA*_2_ genes (Fig. 1A).

The transcriptional organization of *oppDFBCA* and *optABCDF* clusters was confirmed by RT-PCR analyses using oligonucleotides that amplified intergenic regions (Fig. 1C and Supplementary Table 1). RNA extracted from cells grown in CDM was used to generate cDNA, which served as a template for the PCR amplification using specific primer pairs (Fig. 1 and Supplementary Table 1). PCR with primer sets F-oppD/R-oppC and F2-optB/R-OptF yielded products of the expected size (2,643 and 2,241 bp, respectively), indicating that the transcripts extend from *oppD* up to *oppC* (*oppDFBC*), and from *optB* up to *optF* (*optBCDF*), respectively (Fig. 1C). For the *opp* cluster, no RT-PCR product was observed when primer sets F2-oppC/R2-oppA_1_ and F2-oppA_1_/R2-oppA_2_ were used (Fig. 1C). The lack of evidence for co-transcription of *oppA*_1_ with *oppC*, as well as *oppA*_1_ with *oppA*_2_, suggests that the *oppA*_1_ gene is transcribed monocistronically and independently from the *oppDFBC* operon. Similarly, no RT-PCR product was observed when F2-optS/R2-optA and F2-optA/R-optB primer sets were used (Fig. 1B). These results suggest that *optA* gene is transcribed monocistronically while a single transcript encompasses the *optBCDF* genes (Fig. 1). Positive PCR controls were run using the mentioned primer sets and genomic DNA as template; the amplified products were all of the expected size (Fig. 1C). Specific amplicons for *oppA*_1_, *oppA*_2_, *oppC*, *optS* and *optA* genes were detected by RT-PCR analyses by using internal specific primers (data not shown) and no PCR product was detected in any of the negative control reactions (RNA without reverse transcriptase).

### Influence of the peptide supply on the transcription of the proteolytic system

Previously, we demonstrated that PrtL activity was modulated by the peptide content of the growth medium^7^. In order to analyze the effect of the peptide supply on the expression of the complete set of genes involved in the proteolytic system of *L*. *delbrueckii* subsp. *lactis* CRL 581, qRT-PCR experiments were performed for 23 target genes with cDNA obtained from cells grown in CDM with and without Casitone (Table 2). The *prtL* expression was about 115-fold higher in CDM compared to CDM plus Casitone (Table 2). Similarly, the addition of peptides to CDM decreased the transcriptional levels of *oppA*_1_, *oppB*, *optS*, *optA* and *optB* by 29-, 29-, 14-, 5- and 9-fold, respectively (Table 2), while the transcription of *oppA*_2_ was not affected by the presence of Casitone. In general, the influence of the peptide supply on the transcription of peptidase genes was lower than that observed for the other components of the proteolytic system. The expression of eight out of 16 tested peptidase genes (*pepM*, *pepP*, *pepO*, *pepN*, *pepG*, *pepT*, *pepX* and *pepL*) was up-regulated in the presence of peptides (Table 2). On the other hand, no statistically significant differences in the expression level of *pepC*, *pepA*, *pepF*, *pepR*, *pepD*, *pepI*, *pepQ* and *pepV* were observed in the presence or absence of Casitone in the growth medium (Table 2).

**Table 2:** Relative expression ratio of genes related to the proteolytic system of *L*. *delbrueckii* subsp. *lactis* CRL 581 during growth in CDM versus CDM plus Casitone.

Predicted funcion	Gene	Relative expression ratio	*P*-value
Proteinase	*prtL*	115.1	0.0019
Peptide transporters	*oppA*_*1*_	29.45	0.0444
	*oppA*_2_	0.60	0.3900
	*oppB*	28.81	0.0029
	*optS*	13.84	0.0319
	*optA*	5.24	0.0300
	*optB*	9.01	0.0086
Peptidases	*pepM*	3.72	0.0467
	*pepP*	10.14	0.0036
	*pepC*	2.91	0.0585
	*pepA*	1.12	0.4074
	*pepO*	4.39	0.0189
	*pepN*	3.19	0.0135
	*pepF*	1.88	0.2862
	*pepR*	1.73	0.0546
	*pepD*	1.51	0.1520
	*pepG*	1.36	0.0300
	*pepI*	1.50	0.0570
	*pepQ*	1.81	0.0530
	*pepT*	1.41	0.0400
	*pepV*	1.30	0.0541
	*pepX*	6.63	0.0300
	*pepL*	1.52	0.0288

### Proteomic analysis of the peptide supply response

**Figure 2:**
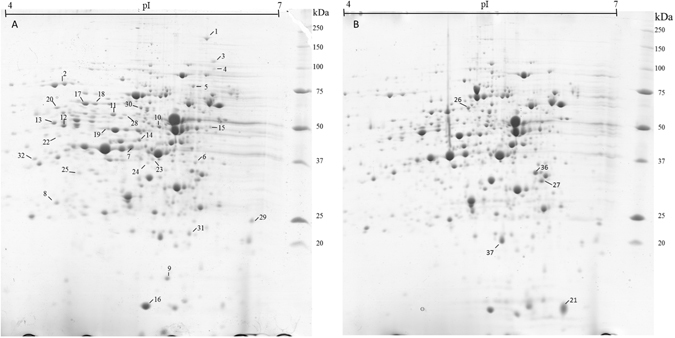
Two-dimensional gels (pH 4 to 7) of the cytoplasmic proteins of *L*. *delbrueckii* subsp. *lactis* CRL 581 grown in CDM (**A**) and CDM plus Casitone (**B**). Gels were stained with Coomassie Brilliant Blue G-250. The protein spots which are differentially regulated in CDM or CDM plus Casitone are indicated. The Precision Plus protein Standard (Bio-Rad) was used as marker.

To better understand the response of *L*. *delbrueckii* subsp. *lactis* to the peptide supply, the proteome of the CRL 581 strain cultivated in CDM and CDM with Casitone were compared. An average of 644 different protein spots was observed in each representative image of two-dimensional gel electrophoresis profiles of cells grown with (treated) or without (control) peptides (Fig. 2). Comparison of the protein patterns revealed that 34 spots displayed differential expression levels (≥2-fold and *P* < 0.05) under both conditions tested (Table 3). Among these, 30 proteins were up-regulated and 4 were down-regulated upon growth in CDM compared to CDM plus Casitone (Fig. 2 and Table 3). In some cases, isoforms of the same protein were identified: i.e., four isoforms of PrtL (spots 1, 3, 4 and 5), three isoforms of the glyceraldehyde-3-phosphate dehydrogenase (spots 11, 19 and 28), three isoforms of the formyl-tetrahydrofolate ligase (spots 17, 18 and 22) and two isoforms of the GTP-binding protein (spots 33 and 34) (Fig. 2 and Table 3).

**Table 3:** Proteome modification of *L*. *delbrueckii* subsp. *lactis* CRL 581 during growth in CDM compared to CDM supplemented with Casitone.

Protein function/Protein name (spot number)	Estimated MM (kDa)	Gene	Fold change	*P* value	Accession number
**Up-regulated**					
Proteolytic system					
Cell envelope-associated proteinase (1, 3, 4, 5)	209.09	*prtL*	7.4	5.6 · 10^−4^	gi|511181292
Aminopeptidase G (10)	49.73	*pepG*	1.8	3.3 · 10^−4^	gi|3024380
Oligopeptide ABC transporter, ATP binding protein (23)	37.53	*oppD*	2.4	1.2 · 10^−3^	gi|511181528
Oligopeptide ABC transporter, ATP-binding protein (32)	36.23	*optF*	1.7	5.0 · 10^−3^	gi|511182605
Amino acid metabolism					
BCAA aminotransferase (7)	37.99	*bcaT*	2.9	7.6 · 10^−7^	gi|511181427
Glutamine/arginine ABC transporter ATP-binding protein (8)	27.74	*glnQ*	1.9	1.0 · 10^−3^	gi|511182879
S-ribosylhomocysteinase (9)	18.08	*luxS*	1.9	1.3 · 10^−2^	gi|511182382
Aromatic amino acid aminotransferase (14)	43.53	*araT*	1.7	1.2 · 10^−4^	gi|325686041
Diaminopimelate epimerase (24)	36.21	*dapF*	2.9	3.0 · 10^−3^	gi|656122331
Nucleotide metabolism					
GMP synthase (30)	57.58	*guaA*	1.5	3.2 · 10^−2^	gi|511182616
Adenylosuccinate lyase (13)	49.17	*purB*	1.7	3.5 · 10^−2^	gi|511181588
Formate-tetrahydrofolate ligase (17, 18, 22)	60.63	*fhs*	1.8	2.4 · 10^−2^	gi|656123743
Stress response					
ATP-dependent Clp proteaset (2)	76.93	*clpx*	1.3	7.0 · 10^−3^	gi|656119779
ATP-dependent protease (31)	21.35	*clpP*	1.6	4.0 · 10^−3^	gi|656120607
Molecular chaperone GroEL (15)	57.31	*groEL*	1.5	6.0 · 10^−5^	gi|511180975
Oxidation/reduction processes					
NAD synthetase (6)	30.83	*nadE*	3.6	8.9 · 10^−5^	gi|511182791
Glutathione reductase (20)	48.49	*gshR*	1.2	7.0 · 10^−2^	gi|656121054
Carbohydrate metabolism					
Glyceraldehyde-3-phosphate dehydrogenase (11, 19, 28)	51.60	*gapN*	1.7	3.0 · 10^−3^	gi|656120154
L-2-hydroxyisocaproate dehydrogenase (25)	33.13	*hicDH*	1.8	4.5 · 10^−2^	gi|511182374
Translation					
30S ribosomal protein S1 (12)	44.32	*rpsA*	1.7	3.1 · 10^−5^	gi|511181889
Vitamin metabolism					
Pyridoxamine 5′-phosphate oxidase (16)	13.16	*pdxH*	1.5	6.2 · 10^−4^	gi|511182483
Miscellaneous					
GTP-binding protein TypA (33, 34)	68.54	*typA*	1.8	9.0 · 10^−3^	gi|656121938
**Down regulated**					
Glutamyl-tRNA synthetase (26)	57.17	*gltX*	0.5	2.5 · 10^−5^	gi|656121195
Glucose-1-phosphate uridylyltransferase (35)	33.91	*galU*	1.4	9.1 · 10^−3^	gi|511182946
DNA-binding regulatory protein (36)	26.53	*yebC*	1.8	8.0 · 10^−3^	gi|511181712
50S ribosomal protein L10 (37)	18.18	*rplJ*	1.3	1.0 · 10^−2^	gi|511180912

The most significant change among all differentially expressed proteins was observed for PrtL (7.4 fold, Table 3), result that was corroborated by SDS-PAGE analysis (Supplementary Fig. S1). SDS-PAGE analysis of CDM grown cells showed a single protein band of approximately 190 kDa corresponding to the expected molecular mass of the proteinase. Contrariwise, this band was not detected in cells grown in CDM with Casitone or MRS (Supplementary Fig. S1, lanes 2 and 3, respectively). The identity of PrtL was confirmed by MALDI-TOF analysis (gi|511181292).

Five enzymes, BCAA aminotransferase (BcaT), aromatic amino acid transaminase (AraT), glutamine/arginine transport ATP-binding protein (GlnQ), S-ribosyl homocysteinase (LuxS), and diaminopimelate epimerase (DapF), involved in the biosynthesis, transport or interconversion of amino acids, were synthesized at higher levels during microbial growth in CDM without peptide supplementation (Table 3). In addition, the up-regulation of enzymes involved in the purine metabolism such as formyl-tetrahydrofolate (THF) synthetase (Fhs), adenylosuccinate lyase (PurB) and GMP synthase/glutamine amidotransferase (GuaA) was observed. Furthermore, several stress response proteins such as chaperonin GroEL, ClpX and ClpP proteases were more abundant during microbial growth without peptides. Concerning the carbon metabolism, the relative amounts of NADP-dependent glyceraldehyde-3-phosphate dehydrogenase (GapN) isoforms were increased during growth in CDM as compared to CDM plus Casitone.

On the contrary, the amount of the 50 S ribosomal protein L10, the protein involved in aminoacyl-tRNA biosynthesis (glutamyl-tRNA synthetase) and the enzyme glucose-1-phosphate uridylyl transferase, which has a central role in galactose metabolism, glycogen synthesis and in the production of glycolipids, glycoproteins, and proteoglycans, were lower in CDM than in CDM plus Casitone. More interestingly, a DNA-binding protein, YebC, was up-regulated in the presence of Casitone.

### Role of YebC on the expression of proteolytic genes

**Figure 3:**
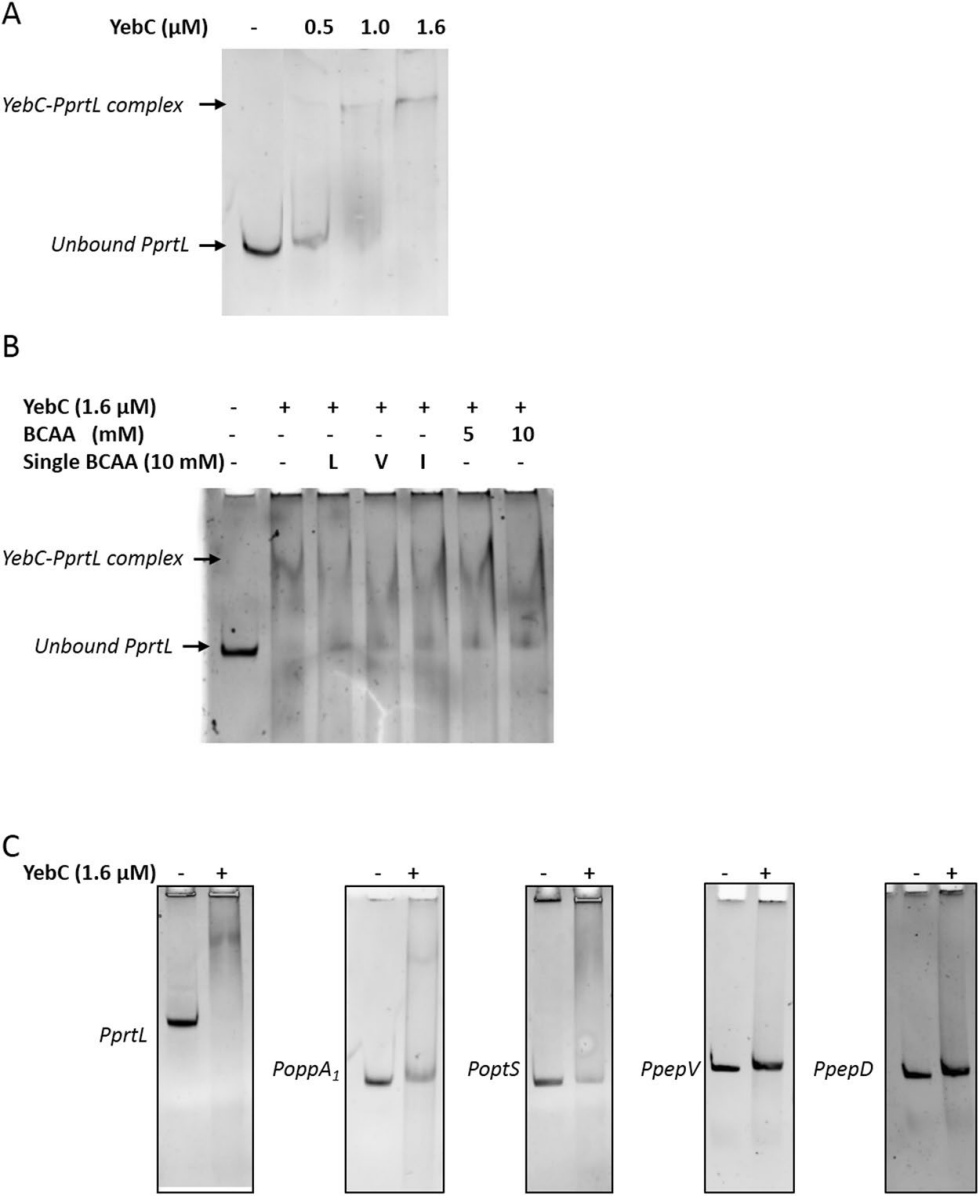
Electrophoresis gel mobility shift assay (EMSA) for YebC binding to different gene promoter regions. (**A**) EMSA assays performed using increasing concentrations of the purified H6-YebC (0, 0.5, 1.0 and 1.6 µM) and 50 ng of DNA corresponding to the *prtL* promoter region (*PprtL*). (**B**) Effect of BCAA on YebC DNA binding to *prtL* promoter region. A DNA fragment containing the *prtL* promoter region (*PprtL* lane 1) was incubated with 1.6 µM of purified H6-YebC in the absence (lane 2) or presence of 10 mM of leucine (L, lane 3), valine (V, lane 4), isoleucine (I, lane 5), 5 mM (lane 6) and 10 mM (lane 7) of each BCAA. (**C**) Binding of YebC to selected promoter regions. 50 ng of target DNA containing the promoter region of *prtL* (*PprtL*), *opts* (*PoptS*), *oppA*_1_ (*PoppA*_1_), *pepD* (*PpepD*) and *pepV* (*PpepV*) were incubated with 1.6 µM of YebC.

To date, no available data exist concerning the direct interaction between YebC and the promoter region of some components of the proteolytic system. For this purpose, the binding ability of YebC to the promoter region of several genes of this system was evaluated (Supplementary Fig. S2) by electrophoresis mobility shift assays (EMSAs) using purified H6-YebC protein (Fig. 3). Firstly, the effect of YebC on the transcriptional expression of *prtL* (the most up-regulated gene in the absence of peptides) was analyzed (Table 2 and Fig. 3A). EMSA analysis revealed that purified H6-YebC was capable of forming a protein-DNA complex with the *prtL* promoter region. This binding was dependent on the YebC concentration (Fig. 3A); the addition of 1.6 µM of purified YebC induced the formation of the YebC-*prtL* promoter fragment complex, as observed by the mobility shift of the *prtL* promoter band (Fig. 3A). Since the presence of BCAA enhanced the BCAAR and CodY (transcriptional regulators of the proteolytic system of *L*. *helveticus* and *Lc*. *lactis*, respectively) affinity for their targets, we examined the effect of isoleucine (I), leucine (L) and valine (V), together or separately, on the formation of the YebC-*prtL* promoter complex (Fig. 3B). Concentrations of up to 10 mM of BCAA did not affect the binding ability of the YebC transcription factor to the *prtL* DNA fragment (Fig. 3B). Besides *prtL*, the expression of several genes of the proteolytic system was regulated by the presence of peptides in the growth medium (Table 2). EMSA assays were carried out to evaluate if the up-regulated *oppA*_1_ and *optS* genes were under direct control of YebC (Fig. 3C). The DNA fragments corresponding to *oppA*_1_ and *optS* were retarded in the presence of 1.6 μM of YebC (Fig. 3C), demonstrating the binding of this transcriptional factor to the promoter region of these genes. On the other hand, no band shift was detected with a DNA fragment encompassing the promoter region of *pepD* or *pepV*. These genes were further used as negative controls since our transcriptional analysis indicated that their expression was not affected by the presence of peptides in the growth medium (Table 2).

## Discussion

The proteolytic system of *Lactococcus* is the best documented among LAB^2, 7, 9^. The lack of knowledge of this system in lactobacilli could be related to the difficulty to genetically modify certain species of this group such as thermophilic lactobacilli. The present study was focused on the proteolytic system of *L*. *delbrueckii* subsp. *lactis* CRL 581, which plays an essential role in bacterial growth contributing to the organoleptic properties of fermented products; additionally, the release of bioactive health-beneficial peptides during milk fermentation can occur^7, 12, 20^. *In silico* genomic analyses suggested that *L*. *delbrueckii* subsp. *lactis* CRL 581 possesses several genes predicted to be involved in casein utilization such as one CEP (*prtL*), two oligopeptide transport systems (*opp* and *opt*) and sixteen intracellular peptidases. *prtL* as well as the peptidase genes have a monocistronic organization while the *opt* and *opp* genes are detected within an operon structure. Transcriptional analysis demonstrated that the *opp* operon consist of four genes *oppD*, *oppF*, *oppB* and *oppC*. In addition, two monocistronic transcripts, *oppA*_1_ and *oppA*_2_ predicted to encode for two peptide-binding proteins were found downstream this operon (Fig. 1). This gene organization was similar to that observed in *L*. *delbrueckii* subsp. *bulgaricus* B14 where duplications of substrate binding proteins in oligopeptide transport systems could be beneficial to the cell^21^. On the other hand, the *L*. *delbrueckii* subsp. *lactis* CRL 581 oligopeptide transport system Opt also has two peptide-binding proteins OptS and OptA. In *Lc*. *lactis*, the *optA* gene is part of the *optABCDF* operon^13^ while in the CRL 581 strain this gene is independently transcribed.

The concentration of essential amino acids and short peptides in milk is very limited; thus, the adaptation of CRL 581 to CDM was analyzed comparing the transcriptional pattern and proteomic profiles of cells cultured in CDM and CDM plus Casitone. CDM was used to mimic the initial growth phase of *L*. *delbrueckii* subsp. *lactis* in milk, where the bacterial growth is independent of casein hydrolysis^22^. Biochemical and genetic studies in dairy LAB such as *L*. *helveticus*, *L*. *delbrueckii* subsp. *lactis* and *Lc*. *lactis* demonstrated that these bacteria reduce the expression of proteinases when short peptides are present in the growth medium^6, 7, 10, 15, 23^. To understand the mechanism underlying the regulation of the proteolytic system in *L*. *delbrueckii* subsp. *lactis* CRL 581, we examined the effect of the peptide supply, by the addition of Casitone to the growth medium, using both transcriptional and proteomics approaches.

The expression of *prtL* was 115-fold repressed when Casitone was present in the growth medium; this result was confirmed by 2DE and SDS-PAGE analysis (PrtL was only observed in the absence of peptides). Transcriptional and proteomic analysis demonstrated that the peptide content of the medium modulates the proteinase biosynthesis in CRL 581 strain.

The addition of peptides to the CDM resulted in the reduced transcription of *oppA*_1_, *optS*, *optA*, *oppDFBC* and *optBCDF*. Furthermore, 2DE gels obtained from cells cultivated in CDM displayed an up-regulation of the soluble components OppD and OptF of the oligopeptide transport machinery Opp and Opt, respectively (Fig. 2 and Table 3). Similarly, the addition of peptides to dough resulted in the reduced transcription of both *dtpT* and *opp-pepN* genes in *L*. *sanfranciscensis* ^24^. In *Lc*. *lactis* as well as in *L*. *helveticus* the peptide content affected the transcriptional pattern of genes encoding proteins of the Opp, and to a lesser extent, Opt transport systems^13, 23, 25, 26^.

Peptidases are relevant in the proteolytic system of LAB since they are involved in the hydrolysis of peptides to release essential amino acids^2, 9^. A study on the aminopeptidase activity of six strains of *L*. *helveticus* grown in milk or MRS medium demonstrated that the mechanism of regulation depended on the specific peptidase^27^. Array data of *L*. *helveticus* CRNZ32 identified several peptidase genes (*pepN*, *pepR*, *pepT*, *pepO*, *pepV* and *pepX*) that were up-regulated in milk versus MRS^26^. In contrast, an up-regulation of genes encoding peptidases PepI, PepQ, and PepD during growth of *L*. *helveticus* CNRZ32 in MRS was observed. In *L*. *delbrueckii* subsp. *bulgaricus* a catabolite control of *pepQ* was demonstrated^28–30^. In our work, the expression of *pepC*, *pepA*, *pepF*, *pepR*, *pepD*, *pepI*, *pepQ* and *pepV* did not seem to be subjected to peptide supply regulation suggesting a role in cellular processes other than in external peptide source utilization. In contrast, the *pepM*, *pepP*, *pepO*, *pepN*, *pepG*, *pepT*, *pepX* and *pepL* expression was dependent on the external peptide source. The transcription repression by nitrogen sources such as Casitone was particularly significant for *pepP* and *pepX*, both proline peptidases that play a special role in casein degradation, due to the high proline content of milk proteins^31, 32^.

The growth of CRL 581 in CDM also led to the up-regulation of stress-related proteins as well as enzymes involved in glycolysis, biosynthesis, interconversion or transport of amino acids, purine metabolism and other functions (Table 3). AraT and BcaT are aminotransferases responsible for the transamination of aromatic and BCAA and methionine, the major precursors of aroma compounds^33^. BcaT and, to a lesser extent AraT, probably regulate the intracellular pool of free BCAA, which is known to be a signal that controls several regulation systems involved in nutrient supply^33^. It has been shown that isoleucine is the effector of transcriptional repression of BCAA biosynthesis in *Lc*. *lactis* ^15, 34^. In addition, intracellular accumulation of BCAA has been identified as the major effector of CodY-dependent repression of the main components of the proteolytic system in *Lc*. *lactis* ^34^. The expression of AraT and BcaT in this microorganism was regulated at a transcriptional level by nutritional factors via the CodY regulator^33^. As in *Lc*. *lactis*, *araT* and *bcaT* from *L*. *delbrueckii* subsp. *lactis* CRL 581 probably belong to the same regulon controlling the expression of some components of the proteolytic system.

Interestingly, a DNA-binding protein belonging to the YebC family was up-regulated in the presence of Casitone. Although the function and biological pathways in which YebC proteins are involved remain elusive, this protein family is widely distributed and may serve as a transcription regulator^35^. Phylogenetic analysis of the *L*. *delbrueckii* subsp. *lactis* YebC protein revealed the presence of homologous proteins in several LAB belonging to the genera *Lactobacillus*, *Lactococcus*, *Leuconostoc*, *Weissella* and *Pediococcus* (Supplementary Fig. S3). In *L*. *delbrueckii* subsp. *bulgaricus* CAUH1, Lbd0677 (a protein belonging to the YebC family, WP_003622770.1) has been described as a potential transcriptional regulator of the acid tolerance response^36^. The predicted binding site of Lbd0677 consists of an eight bp DNA-binding motif (SSTAGACR^36^). The *in silico* analysis of this putative binding site in promoter regions of the differentially expressed genes of the proteolytic system of *L*. *delbrueckii* subsp. *lactis* CRL 581 showed that the consensus motif SSTAGACR was not found in any promoter regions of proteolytic system genes described above (data not shown).

Even though extracellular peptide-dependent repression of proteolytic genes is well documented in Gram-positive bacteria, scarce information is available in lactobacilli. To our knowledge, only two transcriptional regulators related to the proteolytic system of lactobacilli have been described, PrcR and BCAAR from *L*. *casei* BL23 and *L*. *helveticus* CM4, respectively^37, 38^. In *L*. *casei* BL23 PrcR controls a regulon of genes involved in amino acid supply^37^. However, the low response of *prtP* to the presence or absence of tryptone suggests an additional regulatory system acting on this gene^37^. PrcR response is independent of the peptide or BCAA content^37^. On the other hand, no information about proteinase gene expression in response to peptides or BCAA in *L*. *helveticus* CM4 is available^38^. In *Lc*. *lactis* and *Bacillus subtilis*, the main regulation controlling biosynthesis of proteolytic components takes place at the transcriptional level and involves the global pleiotropic CodY repressor that is responsive to BCAA^15, 39^. However, the absence of a CodY-like homologous and CodY box-like DNA sequence at the upstream regions of the proteolytic genes in *Lactobacillus*, suggests a possible role of an unidentified regulatory protein or mechanism able to regulate and coordinate the gene expression of different components of the proteolytic system. Since the protocols to transform *L*. *delbrueckii* subsp. *lactis* were not successful, EMSA assays using purified H6-YebC protein were performed to evaluate the putative role of YebC on the transcription of key genes of the proteolytic system of CRL 581 strain (Fig. 3). These analyses showed that the transcriptional factor YebC negatively regulates the expression of *prtL*, *oppA*1 and *optS* genes. Some other transcriptional regulators, such as the LysR-type, bind target promoters in the absence of their corresponding ligand but require this ligand to activate transcription^40^. Similarly, YebC was able to bind proteolytic promoters regardless the presence of BCAA (Fig. 3)

To conclude, the identification of the regulator YebC acting on the transcription of key components of the proteolytic system in response to peptide supply led to a better understanding on the peptide control and nutritional metabolism in *Lactobacillus*. This finding adds a new piece in the regulation of the proteolytic system of *Lactobacillus* and provides a functional clue for the YebC family proteins.

## Methods

### Bacterial strains and culture conditions

*L*. *delbrueckii* subsp. *lactis* CRL 581 was isolated from a homemade Argentinian hard cheese, and belongs to the culture collection of the Centro de Referencia para Lactobacilos (CERELA-CONICET, Tucumán, Argentina). Working cultures of *L*. *delbrueckii* subsp. *lactis* CRL 581 were propagated twice in MRS broth (Biokar Diagnostics, France) at 37 °C for 16 h. To eliminate carryover nutrients, cells were harvested by centrifugation at 8,000 × *g* for 15 min, washed twice in sterile 0.85% (w/v) saline solution, and resuspended in this solution to the original volume. This cell suspension was used to inoculate i) MRS, ii) a previously formulated chemically defined medium (CDM)^41^, and iii) CDM containing 1% (w/v) Casitone (Difco Laboratories, Sparks, MD), a pancreatic digest of casein consisting of small peptides and amino acids in 4:1 ratio^41^, at an initial optical density (Cary 50; Varian, Inc., Australia) at 560 nm (OD_560_) of 0.07. Cells grown in the different media were harvested by centrifugation (8,000 × *g* for 10 min at 4 °C) at the exponential growth phase (OD_560_ = 0.8).

### RNA isolation and cDNA synthesis

Total RNA was extracted using the Macaloid Clay method outlined by Raya, *et al*.^42^. The concentration of RNA was quantified by using the Qubit® RNA HS Assay Kit (Life Technologies, Buenos Aires, Argentina). The DNA was removed with 5 U of TURBO™ DNase (Life Technologies). The absence of genomic DNA in treated RNA samples was checked by polymerase chain reaction (PCR). cDNA was synthesized from 1 µg of total DNA-free RNA using random hexamer primers and Superscript III reverse transcriptase (Life Technologies) following the manufacturer instructions.

### Quantitative reverse transcription-PCR (qRT-PCR)

Specific primers for 23 genes, corresponding to the proteolytic system of *L*. *delbrueckii* subsp. *lactis* CRL 581 (Supplementary Table 1) were designed using Primer3 Plus software^43^ and their specificity was checked before the quantitative analysis. qRT-PCR was carried out on an iQ5 Real-Time PCR Detection System (BioRad) with the IQ^TM^ SYBR^®^supermix (Bio-Rad) in 96-well plates. Each reaction consisted of 3 µl of a solution of either template DNA or cDNA (10 ng/µl) or water in the non-template controls, 4 µl of primer mix (3 µM of each primer), 3 µl of RNase-free water and 10 µl of IQ^TM^ SYBR^®^supermix (BioRad). Amplification program consisted of a denaturation step at 95 °C for 5 min followed by 40 cycles at 95 °C for 15 s and 62 °C for 30 s. A positive control (DNA), a negative control (total RNA sample) and a non-template control (distilled water) were included in each run. Fluorescence due to the binding of the SYBR Green to double stranded DNA was measured at each cycle and melting curves were performed from 60 to 95 °C (0.05 °C/s) to validate the specificity of the PCR reaction. Gene expressions were normalized by the ΔΔC_T_ method using *recA*, *rpoD* and 16 S *rRNA* as reference genes^44^. A housekeeping gene was used to standardize the expression of a given target gene; then, a ratio between treatments (CDM and CDM plus Casitone) was calculated using the algorithm developed by Pfaffl, *et al*.^44^. Relative expression ratios and statistical analysis were performed using fg Statistics software interface^45^. The cut-off for statistically significant differences was set as *P* value < 0.05.

For transcriptional analysis, RT-PCR was performed by using 1 ng/µl of cDNA in the presence of primers described in Supplementary Table 1. To confirm that no contaminating DNA material was present in the RT-PCR mixture, the RNA sample (200 ng) without reverse transcription reaction was PCR-amplified with specific primers.

### DNA preparation, molecular cloning, and transformation

Standard procedures were used for molecular manipulations^46^. Genomic DNA from *L*. *delbrueckii* subsp. *lactis* CRL 581 was extracted as detailed previously^16^. Restriction enzymes, T4 DNA ligase, and DNA polymerases were purchased from Promega (Buenos Aires, Argentina). PCR amplifications were carried out using GoTaq DNA polymerase (Promega). Plasmids were isolated using spin miniprep kits (Qiagen) and PCR products were purified using Qiaquick purification kits (Qiagen).

### Expression and purification of histidine-tagged YebC

For protein expression and purification, the YebC coding gene was amplified from *L. delbrueckii* subsp. *lactis* CRL 581 chromosomal DNA by PCR using specific primers (Supplementary Table 1). The PCR products were digested with *Bam*HI and *EcoR*I and inserted into the *Bam*HI and *Eco*RI sites of the expression vector pRSETA (Invitrogen). The resulting plasmid was used to transform *Escherichia coli* BL21 (DE3) pLysS; the correct sequence of the insert being confirmed by DNA sequencing and one clone being selected for expression of His-tagged fusion protein. Protein purification was carried out as described previously^47^. Protein fractions were analyzed by SDS-PAGE (Supplementary Fig. S4) and those containing the purified recombinant His-tagged YebC (H6-YebC) protein were concentrated and buffer exchanged (Tris:HCl 100 mM pH 7.5, 100 mM NaCl, 20%, v/v, glycerol) with Amicon Ultra-15 30 K centrifugal filter devices (Millipore). Finally, the amount of protein was determined with a Protein Assay Kit (Bio-Rad Laboratories) using bovine serum albumin (BSA) as the standard protein and the proteins were aliquoted and stored at −80 °C.

### Electrophoretic mobility shift assays (EMSA)

EMSA were carried out according to the previously described method^38^ with some modifications. Binding reactions were carried out in 20 µl of reaction mixture containing 25 mM Tris-HCl (pH 8), 50 mM KCl, 0.5 mM EDTA, 0.01% (p/v) BSA, 1 µg of poly(dI-dC), 10% (v/v) glycerol, 50 ng of PCR fragment, and varying amounts of the purified H6-YebC in the presence or absence of 10 mM of BCAA or 10 mM Leu, Ile, or Val, separately. After incubation at 30 °C for 30 min, protein-DNA complexes were separated in 6% (w/v) polyacrylamide gels and run in TAE buffer at 100 V for 1 h followed by staining with ethidium bromide.

### Two-dimensional gel electrophoresis

Cells grown in the different media were harvested by centrifugation (8,000 × *g*, 15 min, 4 °C) at the mid-exponential growth phase (OD_560_ = 0.8), washed with 50 mM Tris-HCl buffer (pH 7.5) and resuspended to a final OD_560_ of approximately 30 in 50 mM Tris-HCl buffer (pH 7.5) containing 1 mM PMSF and 10 mM EDTA. Cell extracts (CE) were obtained by vortexing the bacterial cell suspensions with glass beads (0.15- to 0.25-mm diameter; Sigma) in a MiniBeadbeater-16 Cell Disrupter (Biospec) for 10 min. Glass bead, unbroken cells and cell debris were removed by centrifugation (14,000 × *g*, 10 min, 4 °C). Protein concentration was determined by the Bio-Rad protein assay (BioRad) and aliquots of 600 μg were stored at −80 °C until the isoelectrofocusing (IEF) assay. Two-dimensional gel electrophoresis (2-DE) was performed as described by O’Farrell^48^. Three protein extracts from CDM and three from CDM plus Casitone cells were run simultaneously. Samples containing 600 μg of protein of *L*. *delbrueckii* subsp. *lactis* CRL 581 were treated with 1 μl of bezonase (Novagen®) at 37 °C for 30 min. Proteins were concentrated by acetone precipitation, centrifuged (15 min, 14,000 × *g*, 4 °C) and then left to air-dry. Protein resolubilization was done in 350 µl of the rehydration buffer and applied by in-gel rehydration method in Immobiline Dry Strip 18 cm, pH 4–7 L (GE Health Care) for 16 h according to the manufacturer’s instructions. The proteins were focused up to 53,500/Vh. Focused IPG gels were equilibrated twice for 15 min in a buffer containing 50 mM Tris-HCl (pH 8.8), 6 M urea, 30% (v/v) glycerol, 2% (w/v) SDS, and 1% (w/v) DTT. For the second equilibration step, DTT was replaced by 2.5% (w/v) iodoacetamide. The equilibrated IPG strips were sealed on top of the SDS-PAGE gel using 0.5% (w/v) agarose. Precision Plus protein Standard (Bio-Rad) was used as molecular mass marker. The second-dimension SDS-PAGE was carried out using the Tris-glycine-SDS buffer system at 10 mA/gel at 15 °C until the dye front reached the bottom edge of the gel. Gels were stained with Coomassie Brilliant Blue G-250^49^, and digitalized using an ImageScanner (GE Health Care) operated by the software, Lab-Scan 3.00 (GE Health Care). Spot detection, quantification, and analysis were performed using the Same Spots software version 4.6.1 (Totallab, UK). Proteins were considered differentially expressed when the spot percentage volume changed more than 1.5-fold with a *P* value < 0.05. At least three biological replicates were performed for each condition. Spots that showed the strongest up- or down-regulation evidence were excised and proteins were submitted to MS analysis.

### Protein identification

Selected protein spots were excised from gels and destained by repeated washing with 50% (v/v) acetonitrile in 25 mM NH_4_HCO_3_. The proteins were then reduced by incubation with 10 mM DTT (1 h, 56 °C), and then alkylated with 55 mM iodoacetamide. Gel plugs were thoroughly washed with NH_4_HCO_3_, dehydrated with 100 µl of 100% acetonitrile, and then dried in a speed-vac. In-gel digestion was performed with 10 ng/μl trypsin (Promega, sequencing grade) in 25 mM NH_4_HCO_3_ at 37 °C for 15 h. Then, the supernatants were collected, and the tryptic peptides were extracted from the gel sequentially with 5% (v/v) trifluoroacetic acid (TFA) at 40 °C for 1 h and with 2.5% (v/v) TFA, 50% (v/v) acetonitrile at 30 °C for 1 h. Extracts were dried in a vacuum centrifuge, re-dissolved in 15 µl of 50% (v/v) acetonitrile, 0.1% (v/v) TFA, and 1 μl of peptide solution was mixed with 1 μl of matrix [4-hydroxy-α-cyanocinnamic acid in 30% (v/v) acetonitrile, 0.1% (v/v) TFA] before spotting onto a MALDI target for co-cristallization. Protein digestion and MS were performed by the proteomic core facility CEQUIBIEM (Buenos Aires, Argentina). All MS data were collected by using an Ultraflex II BukerDaltonic MALDI TOF/TOF equipment. Data were acquired in reflector mode from a mass range of 700 to 4,000 Da, and 1,250 laser shots were averaged for each mass spectrum. Each sample was internally calibrated with trypsin autolysis and keratin peaks. The generated peak list was based on signal-to-noise filtering and an exclusion list. The resulting file was then searched by Mascot with database search parameters including a mass tolerance of 20–100 ppm, one missed cleavage, oxidation of methionine, and carbamidomethylation of cysteine. Only matched proteins with significant scores (*P* < 0.05) were considered. The obtained hit was validated by MS/MS fragmentation of one or two high S/N peaks per sample. All searches were performed against the database of *L*. *delbrueckii* subsp. *lactis* CRL 581 from the annotated genome (http://www.ncbi.nlm.nih.gov/genome/514?genome_assembly_id=167382).

### Phylogenetic Analysis of the YebC Protein

Homologous protein sequences were identified by using BLASTP (cutoff e-value < 10^−60^). All sequence alignments were generated using the Geneious Alignment tool with default settings (Geneious Pro 4.8.5). Phylogenetic trees were constructed using the Geneious Pro PhyML plugin^50^ with the WAG amino-acid substitution model and 1000 boot-strapping.

### Statistical analysis

Statistical analyses were performed with the software package Minitab 14 (Minitab Inc.) using ANOVA General Linear Models followed by a Tukey’s posthoc test, and *P* < 0.05 was considered significant. Unless otherwise indicated, all values were the means of three independent trials ± standard deviation. No significant differences were observed between individual replicates.

## Electronic supplementary material

Supplementary Information
