# Supplementary material for: YebC, a putative transcriptional factor involved in the regulation of the proteolytic system of Lactobacillus
Source: Sci Rep. 2017 Aug 17;7:8579. doi: 10.1038/s41598-017-09124-1 (PMC5561223; doi:10.1038/s41598-017-09124-1)
Supplement: Supplementary file 1 — Supplementary Information [file 41598_2017_9124_MOESM1_ESM.pdf]

Supplementary Information

YebC, a putative transcriptional factor involved in the regulation of the proteolytic system of *Lactobacillus*

Lucía Brown[1], Josefina M. Villegas[2], Mariano Elean[1], Silvina Fadda[1], Fernanda Mozzi[1], Lucila Saavedra[1], and Elvira M. Hebert[1,*]

[1] Centro de Referencia para Lactobacilos (CERELA-CONICET), Chacabuco 145, 4000 San Miguel de Tucumán, Argentina; and **[2]** Instituto Superior de Investigaciones Biológicas (INSIBIO), CONICET-UNT, Argentina

*Address correspondence to Elvira M. Hebert, ehebert@cerela.org.ar

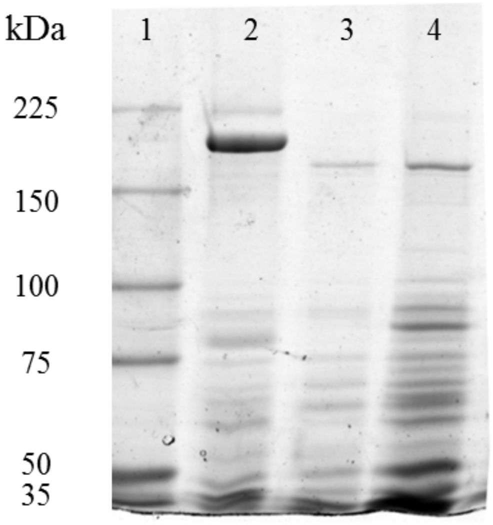

Supplementary Fig. S1. SDS-PAGE of *L. delbrueckii* subsp. *lactis* CRL 581. Lane 1, molecular weight marker (Promega), lane 2, whole cells of *L. delbrueckii* subsp. *lactis* CRL 581 grown in CDM; lane 3, whole cells of *L. delbrueckii* subsp. *lactis* CRL 581 grown in CDM plus Casitone and lane 4, whole cells of *L. delbrueckii* subsp. *lactis* CRL 581 grown in MRS. Gel was stained with Coomassie Brilliant Blue G-250.

prtL

1-AGCTGAGTCAGAGCATGCCGGGGAAATCTGGTACAACATGATCAAAGAGATAGAAGGTTAGAAAGCGGTCAGCAGGTGCTGGCTGCTTTTTCTTTTGCTTTATAAGTTAAACATTTTAATGT

AAATCTAACTTAAAAAACTATAGCTAAAATTTGTCAATATTCTTTTCTTAAATGAAAAAGCTGATAGAACTATGTTTAAATAGGGACGGTGTTATTATAATCAAAAAGGTAACACTATACCACG

ACTTTAGCGAATCAACTTTAAGTTAGTATTTTGTAGATTTTTAACTTCATAGCACGTTATTTTCTCGCGTATTGCTGAAATAATGTGTTTTTTGTTTTTTGGCGTTCAGAAGTTACTTTTCCAC

TGTTTGATTTAAAATTCTGCTAAAAAACACTTAACTTTAATTTAAACTATGGTATGATTATGA**A**ATGTTAGTGAGCTAAAGCTGTGTG**ATG**AAAGGCTTGC-470

oppA₁

1-GAGCATCAACTATGTCGGCCAGGCCCTGCAAAAGGCTGGCGACGCCCGGCAAAGAGAAAACTAAAAACAATTAGCATTAAATTAAAATTATCATTGACTTTTAAAATTAAACTGCTAAAATA

AAAAGCATG**A**AAGATAAAATTTGTGAATCGCTATCTCGATAACTATTCATATGCTGTGTGTTTTATCTTTTGAATATATTGTGTGGAGGTAAATTAAATT**ATG**AAGAGAAGAATGCTCGGAACA

TTTGGAGTACTTCTGGCCGGGGCAGCTTTGCTGGCCGGTTGCGGCAAGAGCAGCTCCAGCTCCAGCAACAGCGGGGCTAAGGATC-331

optS

1-ACTTGGAACTTCAAGGACACTTACATCAAGTAATCAAGCAAAAAGAAGAGGCTCAGCCTCTTCTTTTTTGTGCTATAAGTATTTTTAGATTGACTTGTATCTGTTTTTGGATATTTAGATT

GTTGAAATAAAATAGCAC**T**TTTTCTTAAAAATACTTGAAGAAGCATAAACGAAGAACTATAATGAAAATATCATTAAAGGCTGCTGTAGAGCTTTAATTTGTGTAGGCGCTGGAACAGTCAGT

AAAAGCAAGGAGAAAAAG**ATG**AAATTATCTAAGACTTTAAGCCTGGGAGCCAC-297

pepD

1-GCAAAAGGACCAGCTCCGAAAAAGAGCCGGTCCTTTTTTCAACCGGTATAATCTAGCTATCCCCTATTTTGTATTGTAAAAATCTTTCTCATTTTCCGATAATTTCTT**G**CCCAGCTCCTTCT

TAATAAAGTAGTTTCTCTTTTCGTATAGGATTTTAATTTAACGGTGAGAGGCTTATGATTTCTTTTGGCAAGCGCTAACAAGCACTCCTGTGTGATTGATAGGATTATATTTGATAGGGGGAAA

TATC**ATG**CGTAAGTTTAT-264

pepV

1-AGAGTGATTGATATCGCGGCAACTAAATACCGCCGCTAAAGCTGCCGGAAGGCGGCTTTTTTGCTTGGCGTCTTGTTTGCCAGGGCTGGCGAAGATTGATAGAATTTTAT**T**TAGGTGGTTA

GTTGCTTTTTGTAACTAATTTCAAAAAACAGCAGGCATTGAAAATAAACATTAGGAGAAAAAG**ATG**GACTTAAACTTTAAAGAACTGGCGGAAGCCAA-219

Supplementary Fig. S2. Sequence of *prtL*, *oppA₁*, *optS*, *pepD* and *pepV* DNA fragments used in EMSA assays. Numbers of the first and last residues of the entire fragments used in gel retardation assays are given. Black arrows below the sequence denote the position of the primers used to generate the DNA fragment for EMSA assays. The bent arrow denotes the putative transcription start site (predicted by the Berkeley *Drosophila* Genome Project Neural Network Promoter Prediction *online* program) and the open boxes the putative -10 and -35 regions, respectively, as predicted by Bprom (Softberry, Inc.). Translation-initiation codons are underlined.

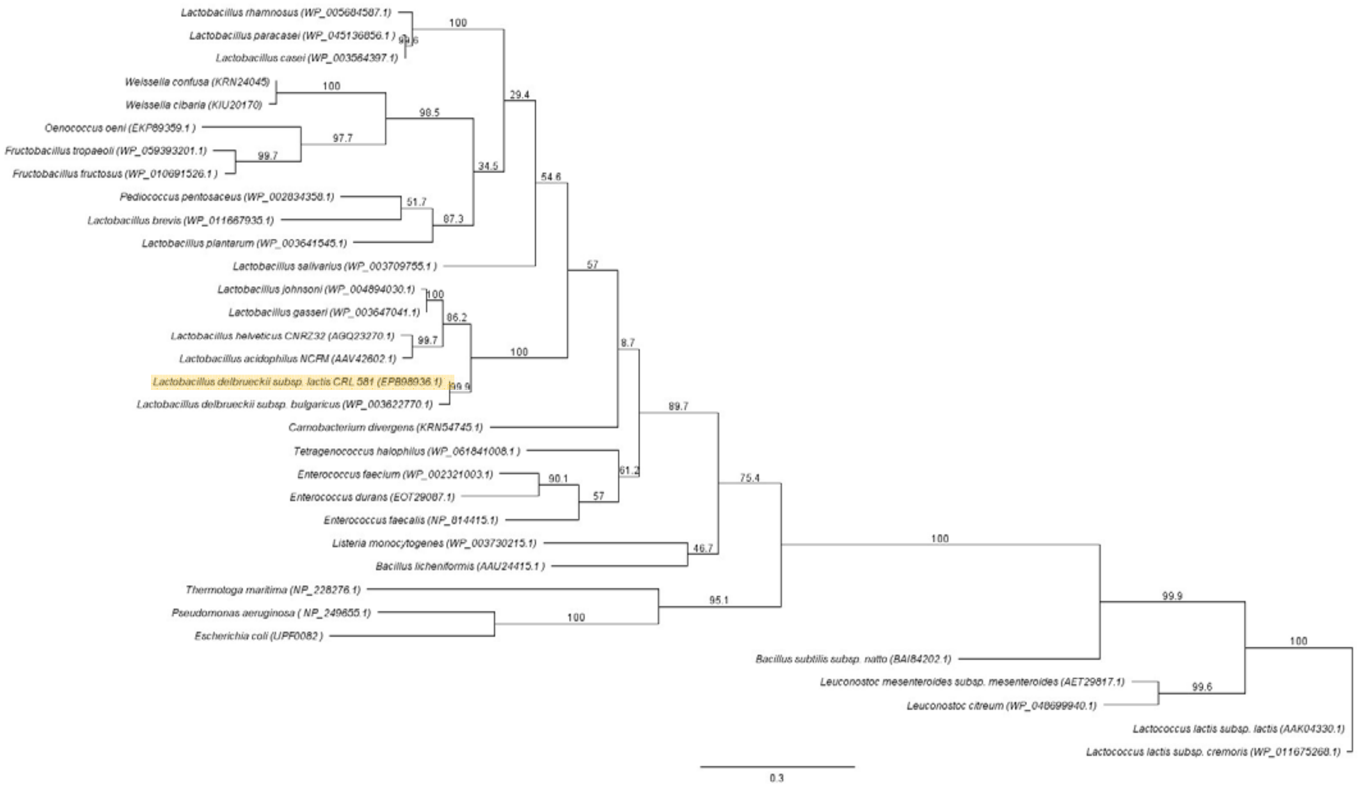

Supplementary Fig. S3. Protein maximum likelihood tree of YebC where the protein of *L. delbrueckii* subsp. *lactis* CRL 581 is highlighted. Maximum likelihood phylogenetic tree was generated using PHYML v2.4.5 (58). GenBank accession numbers are listed after the name. Numbers at the branch nodes are support values from 1,000 bootstrap replicates. The scale bar indicates substitutions per site.

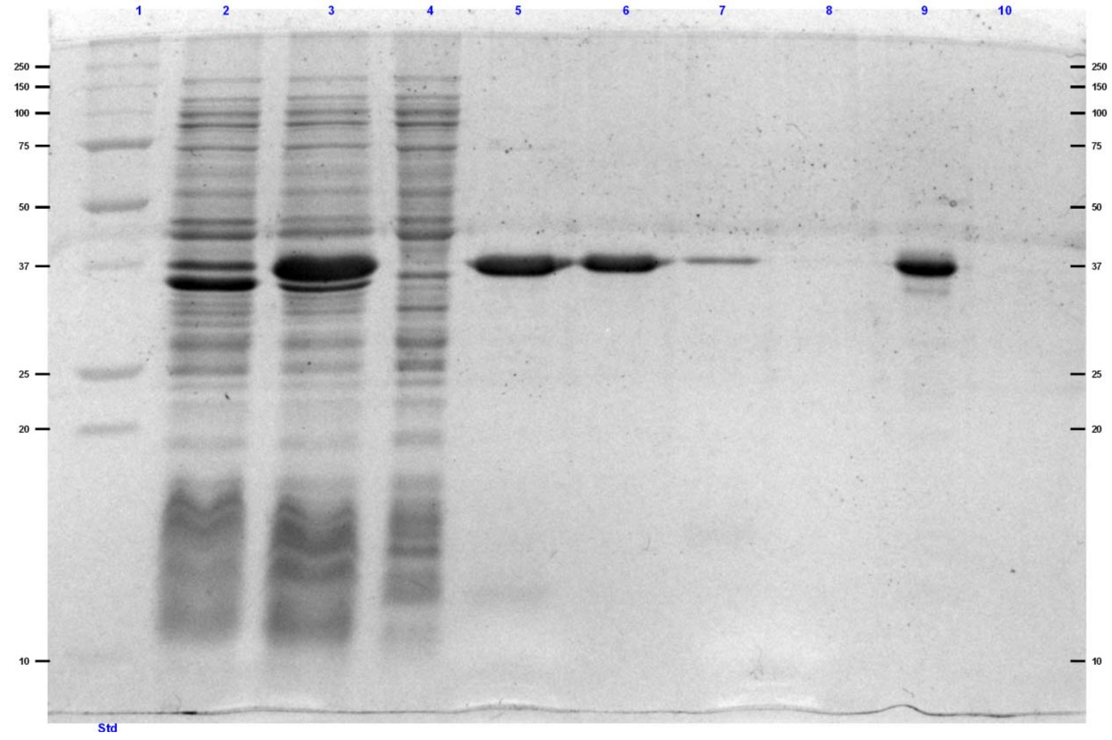

Supplementary Fig. S4. Purification of His-tagged (H6)-YebC. Lane 1, molecular mass markers (Precision Plus Bio-Rad); Lane2, total cytoplasmic extract of *E. coli* BL21 (DE3) pLysS carrying the pRSETA-His-Tagged YebC recombinant plasmid without IPTG induction; lane 3, total cytoplasmic extract of *E. coli* BL21 (DE3) pLysS carrying the pRSETA-His-Tagged YebC recombinant plasmid after 4 h of IPTG induction; lane 4, fraction of total cytoplasmic extract of *E. coli* BL21 (DE3) pLysS carrying the pRSETA-His-Tagged YebC recombinant plasmid applied to a metal chelate affinity-column (Ni-NTA) and washed with buffer containing 10 mM imidazole; lane 5, eluted with buffer containing 50 mM imidazole; lane 6, eluted with buffer containing 100 mM imidazole; lane 7, eluted with buffer containing 200 mM imidazole; and lane 8, eluted with buffer containing 400 mM imidazole.

Gene or target (locus)	Predicted function	Primer sequence (5´to 3’)	Amplicon length (bp)
**qRT-PCR analyses**			
prtL (G134_ RS06625)	Proteinase	F-prtL: CAGCTACAGTCAAGCGAGTGTC<br>R-prtL: TCGCGATCAGATCAGGATCAGT	179
oppA$_1$ (G134_RS05820)	Oligopeptide binding protein	F-oppA$_1$: ATCTGACACGCCAGTCAAGG<br>R-oppA$_1$: GCCTTCCGGTCCATCTTGAA	159
oppA$_2$ (G134_RS05825)	Oligopeptide binding protein	F-oppA$_2$: CCGCAAGGTCGTGATTCACT<br>R-oppA$_2$: GATGCAGGTTCTCAGCTTA	133
oppB (G134_RS05810)	Permease protein	F-oppB: GGCTTTACTCTGGGCTGGTT<br>R-oppB: ATCCCGGTTCGCAGGTATTG	161
opts (G134_RS01500*)*	Peptide binding protein	F-optS: TTGACCGCCAAGGACTTTGT<br>R-optS: GCTTGTACTTGCCAACAGCC	163
optA *(*G134_RS01475*)*	Peptide binding protein	F-optA: CTCTACTGGCTGGGCGAAAA<br>R-optA: GCGCCAGGCATAAACGAAAT	159
optB (G134_RS01470)	Permease protein	F-optB: GCAGCACAGACGATCATGCC<br>R-optB: CTTCGATCCGGCTAAGACCC	145
pepN (G134_RS03335)	Aminopeptidase	F-pepN: AGCCTACCTGCTTTTGGACC<br>R-pepN: TCGTTCAGCCAGAGGTTGTC	141
pepC (G134_RS08320)	Aminopeptidase	F-pepC: TTGAGCAAGCACCCAGCTTA<br>R-pepC: GCCTGACTGCTTTTGGTTGG	156
pepA (G134_RS00305)	Glutamyl aminopeptidase	F-pepA: GGGATTCCTTACACCCGGTC<br>R-pepA: AGCTGCTGAAGCAGTATGGG	136
pepM (G134_RS00100)	Methionine aminopeptidase	F-pepM: ATCGACCAAGCCGTAGTTGG<br>R-pepM: ATGGATGGCTGGATGCCGTGG	125
pepL (G134_RS06955)	Leucine aminopeptidase	F-pepL: TTGGTTGCCAAGCTCTACTC<br>R-pepL: GTCAGCTTGCCAACCATCAT	136
pepG (G134_RS01440)	Aminopeptidase	F-pepG: TGCCGATGGAATACCTGGAG<br>R-pepG: GTCAGCCTTGGACAGGCTAA	172
pepF (G134_RS03600)	Oligopeptidase	F-pepF: CGCGTCAACTTTTGCCTCAA<br>R-pepF: GACGTCGACAGGGATGTTGT	124
pepO (G134_RS01630)	Oligopeptidase	F-pepO: GACGTGGCCTCATCTCTGTC<br>R-pepO: GTCTGCCCGTAGTAGACGCC	158
pepP (G134_RS05655)	Prolidase	F-pepP: ATATAACGGCTACACCGCCG<br>R-pepP: GTCAACGTCCTTGCCAGTTG	148
pepR (G134_RS08875)	Proline iminopeptidase	F-pepR: GGCAACCACGAATACTGGGA<br>R-pepR: CGGCTGGTCAGAGTAAAGGG	118

pepD (G134_RS03315)	Dipeptidase	F-pepD: CTGGTTGCCTGGTGAACATA<br>R-pepD: TTACCGCCACACCTGTTAAG	134
pepV (G134_RS08405)	Dipeptidase	F-pepV: CTCACCAGACGCTGAATACC<br>R-pepV: AACCTGCGGAGTCACGTTAG	141
pepT (G134_RS01035)	Tripeptidase	F-pepT: TTCCTGACCTGCTGACCTTA<br>R-pepT: GGTGCTCATGGAGATACTTG	126
pepX (G134_RS05545)	x-Prolyl-dipeptidyl aminopeptidase	F-pepX: TCCGGCTAAGTCAACTGTGT<br>R-pepX: CCAGTTCGTCGACCTAGAAT	146
pepI (G134_RS08815)	Prolyl aminopeptidase	F-pepI: AGCACGCCATCAAGCTTACG<br>R-pepI: AGCGGTCAGTGTACTCATAG	143
pepQ (G134_RS07685)	Prolidase	F-pepQ: CCAAGCATGAACACGGTACA<br>R-pepQ: GTCCGGTTGACTTCGTAGAT	145
rpoD (G134_RS06440)	RNA polymerase sigma factor RpoD	F-rpoD: TGACTACCAGCTGGGCTTCAAGTT<br>R-rpoD: TTCCACCATGTGAACCGGGATTCT	119
recA (G134_RS05425)	Recombinase RecA	F-recA: CTTGGTCTTGGCAATCGTCC<br>R-recA: AGGTGAAATGGGTGACTCCC	93
16S rRNA (G134_RS08275)	16S ribosomal RNA subunit	F-16Srna: GCCACTGGTGTTCTTCCATA<br>R-16Srna: GCAGGCGGAATGATAAGTCT	146
**Transcriptional analyses**			
oppDFBC		F-oppD: TTGCTTTACCATACGGATATGA<br>R-oppC: AGCGGATGGTGGAATTTGGG	2643
oppC-A		F2-oppC: ATCGTCGACTTCGTGCAGAT<br>R2-oppA$_1$: TCATCGGTTGCAAACAGACC	948
oppA$_1$-A$_2$		F2-oppA$_1$: CCAGCAATGGAAGAAGATCG<br>R2-oppA$_2$: GATGCAGGTTCTCAGCTTA	1254
optBCDF		F2-optB: ACAGTCTGATCGACCCACGGA<br>R-optF: CCCTTGGTGACGGTGCGTCC	2241
optS-A		F2-optS: TCGCTGACTCCAAGACTACT<br>R2-optA: GGTATTGGCGATCTGTTCCT	1930
optA-B		F-optA CTCTACTGGCTGGGCGAAAA<br>R-optB CTTCGATCCGGCTAAGACCC	2499
**Preparation of His-Tagged fusion protein**			
yebC (G134_RS05065)		Yebc-F: CAGAAAGAAGGATCCTTAATG<br>Yebc-R: CCGGAATTCGCTTTCCACAGAAAGTCGCGT<br>Restriction enzyme sites are underlined	804

Supplementary Table 1. Primers used in this study
